# TAF15 promotes cell proliferation, migration and invasion of gastric cancer via activation of the RAF1/MEK/ERK signalling pathway

**DOI:** 10.1038/s41598-023-31959-0

**Published:** 2023-04-10

**Authors:** Li Tang, Chengming Guo, Xu Li, Bo Zhang, Liuye Huang

**Affiliations:** Department of Gastroenterology, The Affiliated Yantai Yuhuangding Hospital of Qingdao University, Yantai, Shandong People’s Republic of China

**Keywords:** Cancer, Biomarkers, Gastroenterology, Oncology

## Abstract

TATA-box-binding protein-associated Factor 15 (TAF15), a member of the FUS/EWS/TAF15 (FET) family, contributes to the progression of various tumours. However, the role and molecular mechanism of TAF15 in gastric cancer (GC) progression are still unknown. In this study, we found that TAF15 was significantly upregulated in GC tumour tissues and cell lines. Overexpression of TAF15 was associated with a larger tumour size, high pathologic stage and high T stage of GC. TAF15 knockdown suppressed the proliferation, migration and invasion of GC cells in vitro and inhibited the tumour growth in vivo. Additionally, TAF15 knockdown led to the significant reductions in the phosphorylation levels of RAF1, MEK and ERK1/2, while total RAF1, MEK and ERK1/2 exhibited no significant change in GC cell lines. In summary, TAF15 is overexpressed in GC tumour tissues and cell lines, and promotes cell proliferation, migration and invasion in GC via the RAF1/MEK/ERK signaling pathway, which suggests that TAF15 might be a potential molecular diagnostic marker or therapeutic target for GC.

## Introduction

Gastric cancer (GC) is a common disease worldwide. Due to its high incidence and mortality, GC is the fourth most common malignancy and the fourth main cause of cancer-related death globally^1,2^. Although the incidence and mortality of GC have declined recently, a larger number of patients can be seen in the future because of ageing populations^3^. In recent years, despite advances in surgical or targeted chemotherapy for GC, the treatment is still not satisfactory due to difficulty in early diagnosis^3–5^. Therefore, identifying novel early diagnostic markers or therapeutic targets is of great importance for the diagnosis and treatment of GC.

TAF15 (TATA-box-binding protein-associated Factor 15) is a kind of RNA or DNA binding protein that belongs to the FUS-EWS-TAF15 (FET) family of proteins, which are involved in RNA splicing, transcription, mRNA transport, signalling, modification, translation and maintenance of genome integrity^6–9^. FET proteins are mainly present in the cell nucleus^10^, however, they are also present in the cell surface and cytoplasm^11,12^. Therefore, they have an extended function, including regulation and interaction with a number of various proteins^9,13–15^. FUS (fused in sarcoma/translocated in liposarcoma, FUS/TLS), a member of the FET family, can interact with lncRNA-MIR137HG and ultimately promote GC progression^16^. Previous studies indicated that chromosomal translocation of FET genes can lead to fusion oncoproteins in several types of tumours including sarcoma and leukaemia^17^. Moreover, TAF15 knockdown affects the expression of a large subset of genes, of which a significant percentage is involved in the cell cycle and cell death^18^. Accumulating studies have shown that TAF15 is involved in the processes of human cancers, including extraskeletal myxoid sarcomas, leukaemia, human neuroblastoma, and lung squamous cell carcinoma^18–21^. In addition, LncRNAs or circRNAs can combine with TAF15 to stabilize downstream target genes such as SMAD3 mRNA, SOCS3 mRNA, and HMGB3 mRNA to facilitate cell proliferation and migration^9,15,22^. Moreover, lncRNA GAS5 binding to TAF15 could increase HIF1A expression, resulting in promotion of wound healing in diabetic foot ulcers^23^. A study has reported that the human antibody PAT-BA4 recognizes a tumour-specific TAF15 antigen that inhibits tumour cell adhesion and spreading in stomach cancer and melanoma^8^. Overexpression of TAF15 is correlated with worsened survival in non-small cell lung cancer patients^13^. Additionally, TAF15 is involved in the drug tolerance of several cancer cells^24^. Most importantly, a recent study has shown that TAF15 can enhance the MAPK6 (mitogen-activated protein kinase 6) expression to activate the MAPK signalling pathway in Lung squamous cell carcinoma^20^. However, to the best of our knowledge, the role and exact molecular mechanism of TAF15 in GC progression have not yet been reported.

In this study, we detected the expression of TAF15 in GC tissues and cell lines. Moreover, our study further explored the role and the underlying molecular mechanism of TAF15 in GC progression. The results identified that overexpression of TAF15 might promote cell proliferation, migration and invasion by activating the RAF1/MEK/ERK signalling pathway in GC. Thus, TAF15 could be a novel target for the diagnosis or treatment of GC.

## Results

### TAF15 is overexpressed and correlates with poor prognosis in GC patients

**Figure 1:**
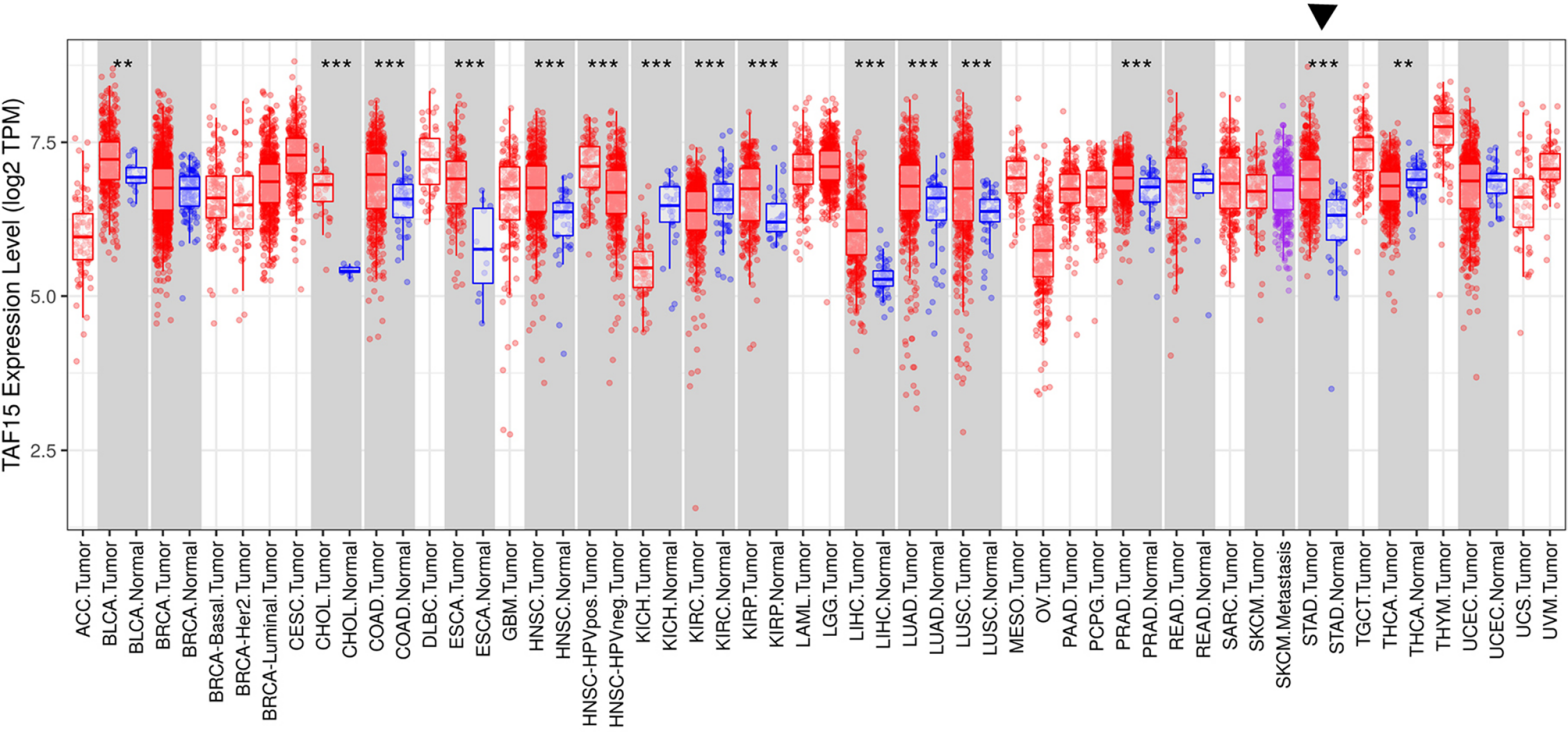
The mRNA expression of TAF15 on a pan-cancer level. The mRNA expression of TAF15 was upregulated in 12 cancer types and downregulated in 3 cancer types from TIMER databases. p values were calculated by student-t test. **p < 0.01, ***p < 0.001.

**Figure 2:**
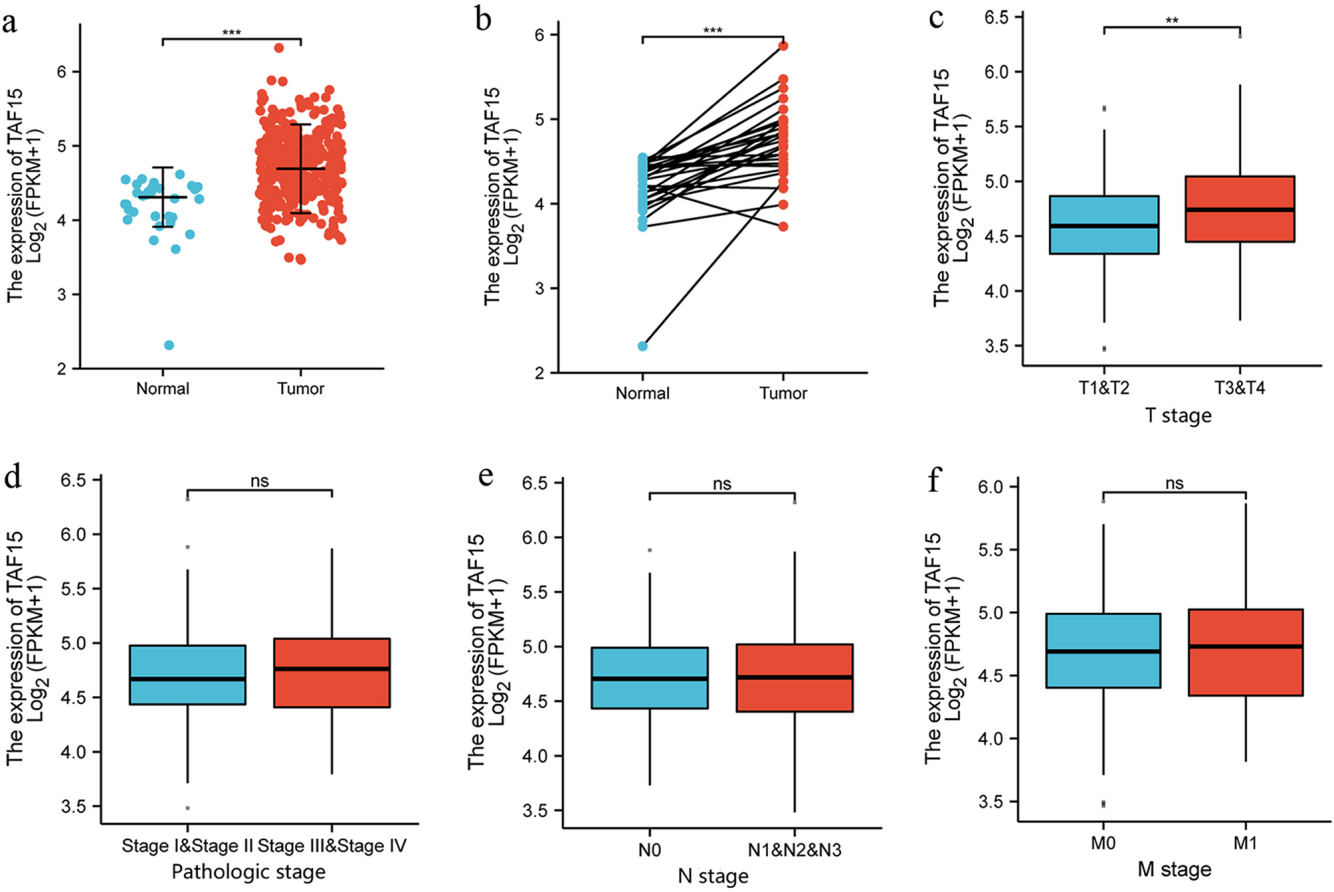
The mRNA expression of TAF15 is overexpressed in GC tissues and correlates with poor prognosis in GC patients. (**a**) The mRNA expression levels of TAF15 in 375 GC samples and 32 adjacent normal samples from TCGA databases. (**b**) The mRNA expression levels of TAF15 in 32 GC tissues and matched adjacent normal tissues from TCGA databases. (**c**) Higher expression levels of TAF15 were identified in GC patients with high T stage. (**d–f**) No significant difference were identified among the expression levels of TAF15 and pathologic stage, N stage, M stage. (**a,b**) p values were calculated by student-t test. (**c**–**f**) p values were calculated by Kruskal–Wallis test. **p < 0.01, ***p < 0.001. ns means no significant.

**Figure 3:**
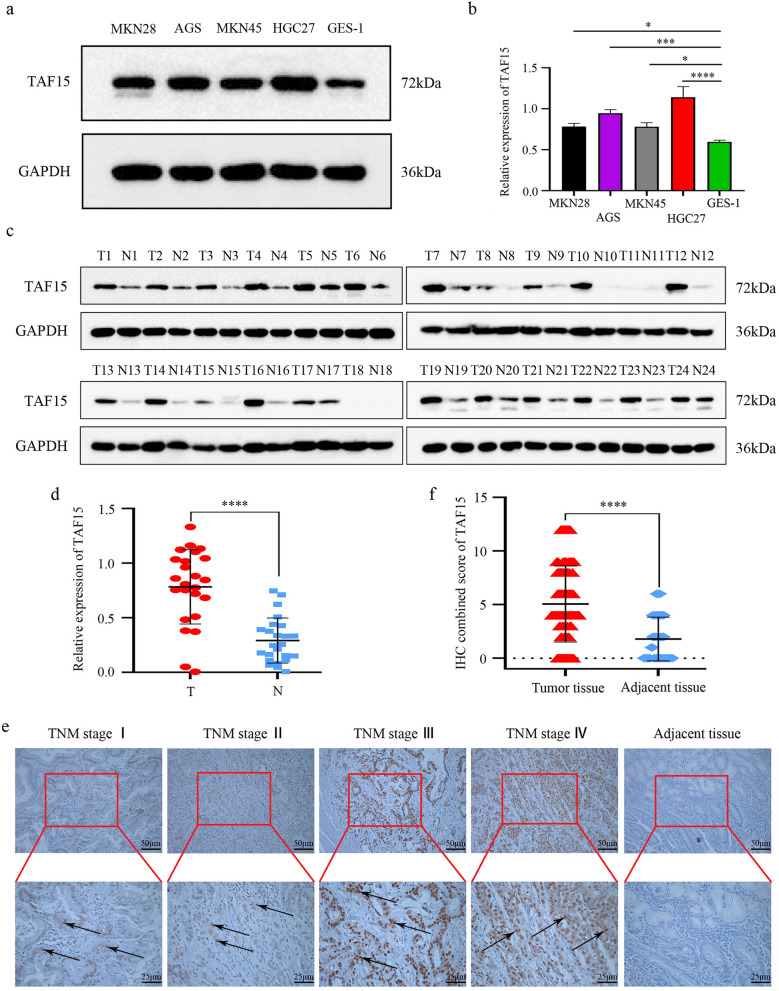
The protein expression of TAF15 is upregulated in GC. (**a,b**) Western blot analysis for the expression of TAF15 in GC cell lines (MKN28, AGS, MKN45, HGC27) and GES-1 (Human Gastric mucosal Epithelial Cell). p values were calculated by One-way ANOVA. The experiment was repeated three times. (**c,d**) Western blot analysis for the expression of TAF15 in 24 GC tissues (T) and matched adjacent normal tissues (N). p values were calculated by student-t test. Three replicate experiments were performed. (**e**) Immunohistochemistry analysis for the expression of TAF15 in 131 GC samples and 24 adjacent normal samples. Representative images are shown. TFA15 staining was mostly detected in cell nucleus, and TFA15 was highly expressed in GC tissues. (**f**) The combined staining score of TAF15 in tumor tissues comparing with adjacent normal tissues. p values were calculated by student-t test. *p < 0.05, ***p < 0.001, ****p < 0.0001.

First, the TAF15 expression level was analyzed from a pan-cancer perspective on the basis of TCGA and TIMER databases. The results indicated that the mRNA expression levels of TAF15 were significantly upregulated in 12 cancer types and downregulated in 3 cancer types (Fig. 1). Subsequently, unpaired data analysis revealed that the mRNA expression levels of TAF15 in GC tissues (n = 375) were significantly higher than those in adjacent normal tissues (n = 32) (Fig. 2a). Moreover, the paired data analysis indicated that the mRNA expression levels of TAF15 in GC tissues (n = 32) were also significantly higher than those in matched adjacent normal tissues (n = 32) (Fig. 2b). These findings demonstrate that TAF15 is overexpressed in GC patients. Then, the Kruskal–Wallis test was performed to evaluate the relationship between the mRNA expression level of TAF15 and the clinicopathological features of GC patients. The results showed that a higher expression level of TAF15 was identified in high T stage GC patients (Fig. 2c), while there was no significant difference in pathologic stage (Fig. 2d), N stage (Fig. 2e) or M stage (Fig. 2f). Second, western blotting analysis was performed on GC cell lines (MKN28, AGS, MKN45, HGC27, GES-1 as control) and 24 paired GC tissues to confirm the expression level of TAF15 in GC. The results indicated that the expression levels of TAF15 in MKN28, AGS, MKN45 and HGC27 cells were significantly upregulated compared to those in GES-1 cells (Fig. 3a,b). Moreover, as shown in Fig. 3c and d, most GC tissues (22 of 24, 91.7%) presented significantly higher TAF15 levels than matched adjacent normal tissues. Furthermore, immunohistochemical analysis was used to further confirm the expression level of TAF15 in a larger range of GC patients, including 131 GC tissues and 24 adjacent normal tissues. The results demonstrated that the combined staining score of TAF15 protein was significantly higher in GC tissue sections that in adjacent normal tissue sections (Fig. 3e,f and Supplemental Table S1, black arrows point to the brown area indicate high expression of TAF15 protein). Moreover, as shown in Fig. 3e, the staining of TAF15 protein in TNM stage I and TNM stage II GC was weaker than that in TNM stage IIII and TNM stage IV GC. Subsequently, we evaluated the relationship between the expression level of TAF15 and the clinicopathological characteristics of GC patients based on the results of immunohistochemical analysis. As shown in Table 1, TAF15 was significantly overexpressed in patients with a larger tumour, high pathologic stage and high T stage, while there was no significant difference among gender, age, differentiation and N stage.

**Table 1:** Correlations between TAF15 expression and clinicopathological characteristics in GC patients (n = 131).

Parameters	TAF15 expression		P value
	Negative	Positive	
Total number	38	93	
Gender			
Male	27	73	0.3631^+^
Female	11	20	
Age (year)			
≥ 60	22	63	0.2839^+^
< 60	16	30	
Tumor size			
Maximum diameter (cm)			
≥ 5	6	75	＜0.0001^++^
< 5	32	18	
Differentiation			
Poor	17	43	0.8758^++^
Moderate/high	21	50	
Pathologic stage			
I + II	23	33	0.0086^++^
III + IV	15	60	
T stage			
T1 + T2	18	20	0.0031^++^
T3 + T4	20	73	
N stage			
N0	16	25	0.0882^++^
N1 + N2 + N3	22	68	

*TAF15* TATA-box-binding protein-associated factor 15, *GC* gastric cancer.

^+^p values were calculated by Chi-square test.

^++^p values were calculated by Mann–Whitney *U* test.

Overall, the results of the online database analysis, western blotting and IHC analysis indicate that TAF15 is overexpressed and correlates with poor prognosis in GC patients.

### Knockdown of TAF15 inhibits cell proliferation, migration and invasion of GC in vitro

**Figure 4:**
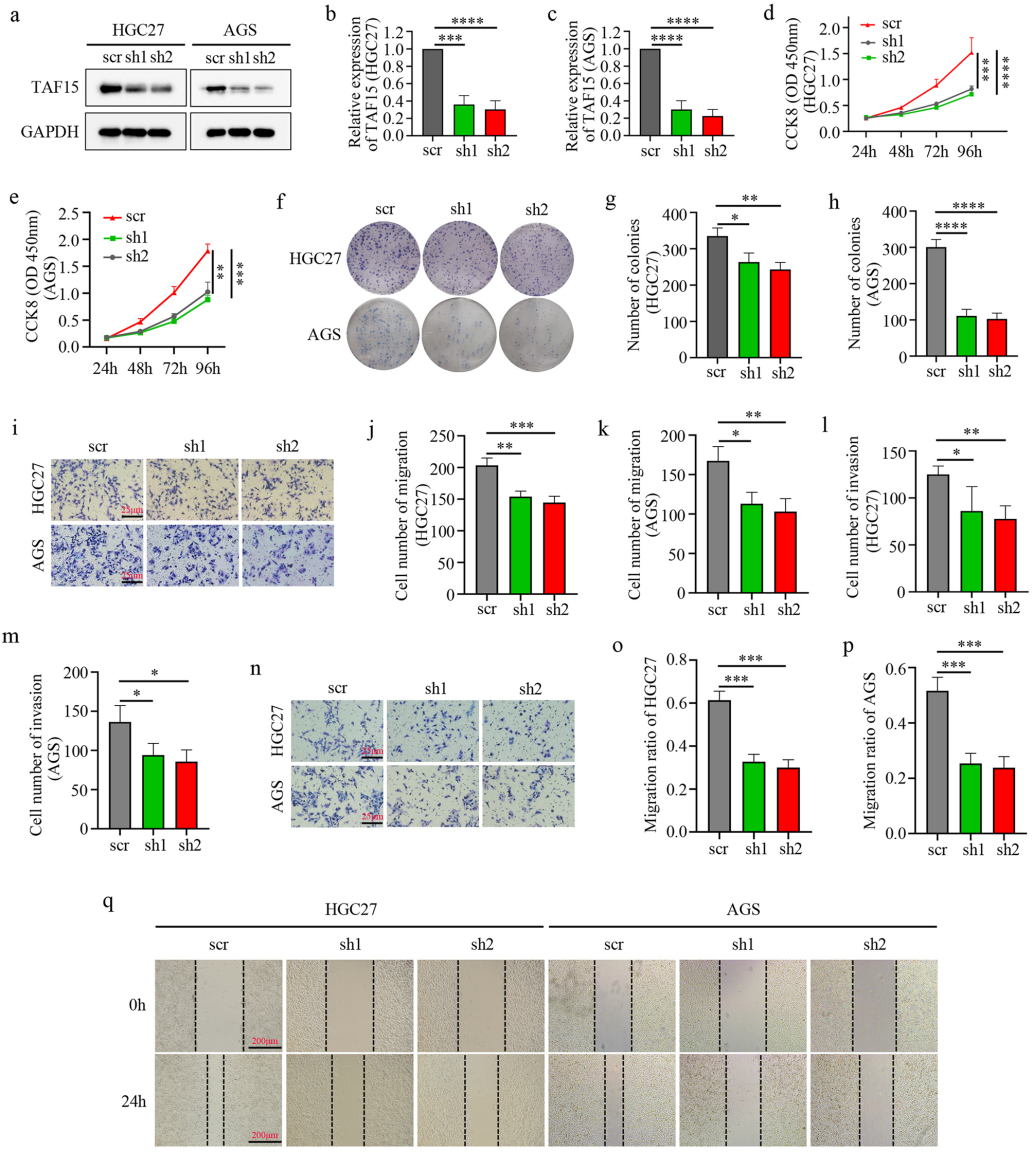
Knockdown of TAF15 inhibits the cell proliferation, migration and invasion of GC in vitro. (**a–c**) The knockdown efficiency of TAF15 shRNA in HGC27 and AGS cells. Knocked down TAF15 by use of two shRNAs (sh1 and sh2) and scrambled shRNA (scr) as control in AGS and HGC27 cells. TAF15 expression level was significantly downregulated in sh1 and sh2 group when compared to scr group as detected by western blotting. p values were calculated by One-way ANOVA. (**d,e**) CCK-8 assay detected the proliferation of AGS and HGC27 after cell transfection. (**f–h**) Colony formation assay detected the proliferation of AGS and HGC27 after cell transfection. (**i–n**) Transwell assay was conducted to analyse the migration and invasion of AGS and HGC27 after cell transfection. (**o–q**) Wound healing assay detected the migration of GIST-882 and GIST-T1 after cell transfection. p values were calculated by One-way ANOVA. *p < 0.05, **p < 0.01, ***p < 0.001, ****p < 0.0001. All these experiments were repeated 3 times.

To reveal the role of TAF15 in GC, we used short-hairpin RNAs (shRNAs) in a lentiviral vector to knock down TAF15 in AGS and HGC27 cells due to the expression levels of TAF15 in these two cell lines were higher than in other cell lines (Fig. 3a,b). Two shRNAs targeting TAF15 (sh1, and sh2) were used to knock down TAF15 and sh-scrambled (scr) was used as a control. The results of western blotting analysis indicated that TAF15 expression levels in the sh1 group and sh2 group were significantly lower than those in the scr group (Fig. 4a–c). Thus, sh1and sh2 were applied for the further study. CCK-8 and colony formation assays indicated significantly lower proliferation (Fig. 4d,e) and colony formation (Fig. 4f–h) in the sh1 group and sh2 group than in the scr group in AGS and HGC27 cell lines. Moreover, transwell and wound-healing assays also showed that the sh1 group and sh2 group exhibited significantly weaker migration (Fig. 4i–k,o–q) and invasion (Fig. 4l–n) abilities than the scr group in AGS and HGC27 cell lines. Based on the above studies, the data confirm that TAF15 knockdown significantly suppress GC cell proliferation, migration and invasion.

### TAF15 regulates the RAF1/MEK/ERK signalling pathway in GC cells

**Figure 5:**
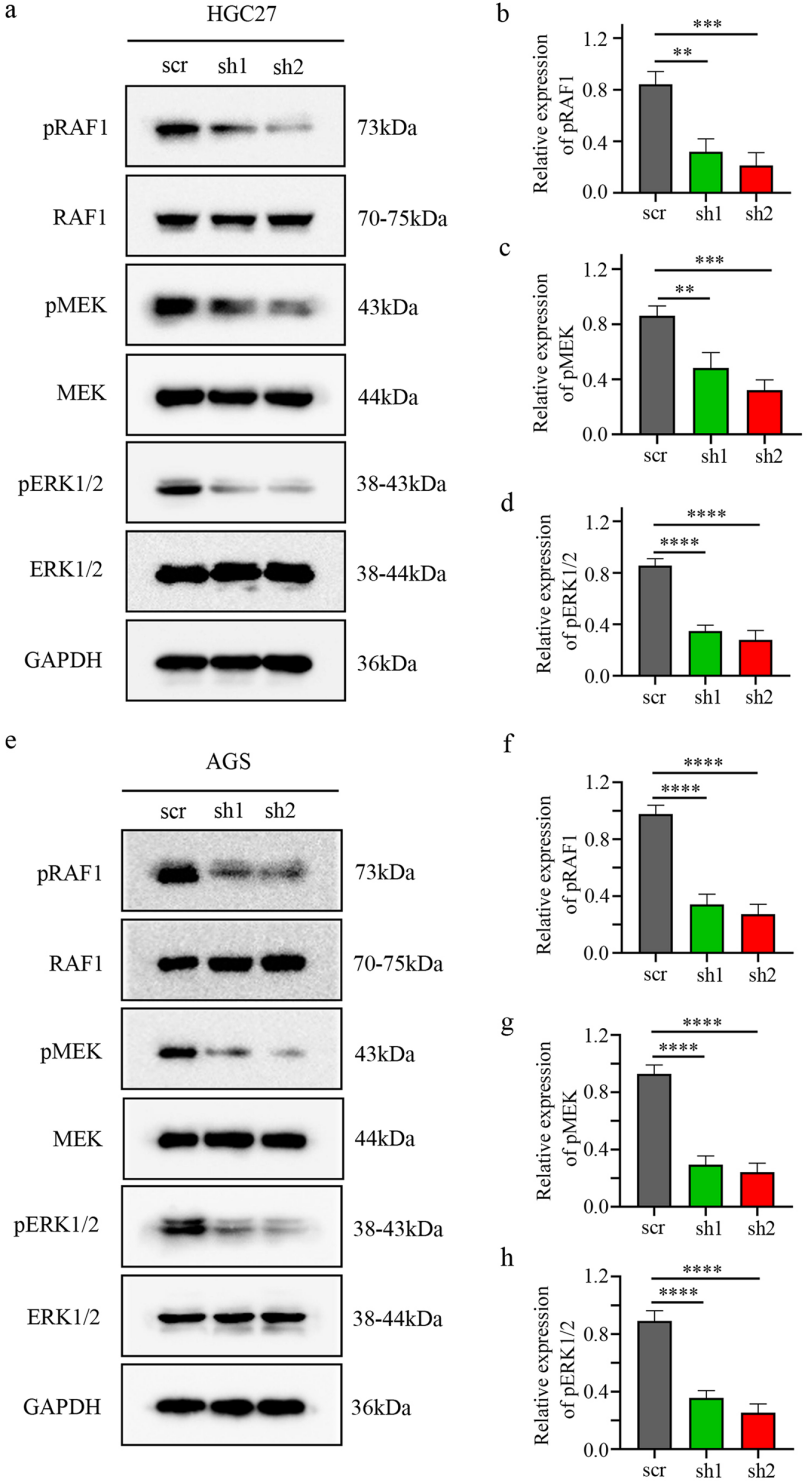
TAF15 promotes the proliferation, migration and invasion of GC cells via the activation of RAF1/MEK/ERK pathway. (**a–d**) Western blotting analysis detected the expression levels of pRAF1/RAF1, pMEK/MEK and pERK/ERK in HGC27 cells after cell transfection. (**e–h**) Western blotting analysis detected the expression levels of pRAF1/RAF1, pMEK/MEK and pERK/ERK in AGS cells after cell transfection. p values were calculated by One-way ANOVA. **p < 0.01, ***p < 0.001, ****p < 0.0001. The experiment was repeated three times.

One study found that EWSR1 (EWS RNA binding protein 1), a member of the FET family of proteins, forms the fusion oncoprotein EWS-FLI1, which promotes the progression of Ewing sarcoma tumours through activation of the ERK1/2 signalling pathway in zebrafish^25^. Moreover, the RAF/MEK/ERK signalling pathway plays a crucial role in the regulation of proliferation, migration, invasion, differentiation, the cell cycle and apoptosis in several types of human cancers, including colorectal cancer, renal cell carcinoma, and hepatocellular carcinomas^26^. Taken together, these data hypothesized that TAF15 might be involved in the regulation of cell proliferation, migration and invasion via the activation of the ERK signalling pathway in GC. Thus, the phosphorylation levels of ERK1/2 were first investigated in AGS and HGC27cells by Western blotting. The results showed that the phosphorylation level of ERK1/2 significantly decreased in the sh1 and sh2 groups, while the total level of ERK1/2 was not significantly changed (Fig. 5a,d,e,h). Subsequently, the phosphorylation levels of RAF1 and MEK were detected in AGS and HGC27cells by Western blotting. The results indicated that the phosphorylation levels of RAF1 and MEK also decreased significantly in the sh1 and sh2 groups, but the total levels of RAF1 and MEK were not significantly changed (Fig. 5a–c,e–g). Overall, these data suggest that TAF15 promotes the proliferation, migration and invasion of GC cells by activating the RAF1/MEK/ERK signalling pathway.

### Knockdown of TAF15 suppresses tumour growth in vivo

**Figure 6:**
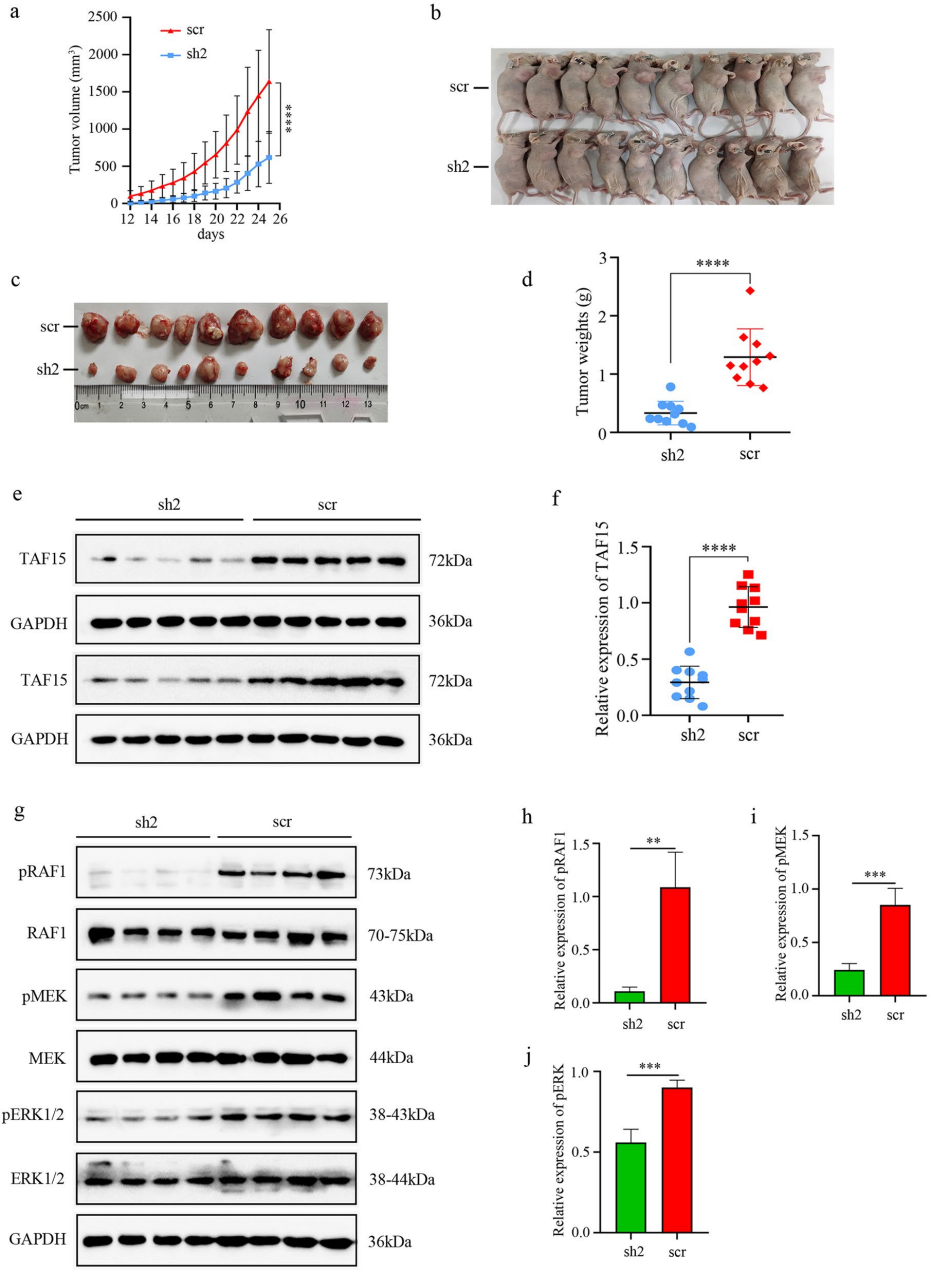
Knockdown of TAF15 inhibits tumour growth of GC in vivo. (**a,b**) Xenograft tumours were obtained (n = 10) when nude mice were sacrificed by using the cervical dislocation method on day 25, and tumor volume was measured every day and a growth curve was drawn. The volumes of xenograft tumours in sh2 group were significantly smaller compared to the scr group. p value was calculated Kruskal–Wallis test. (**c,d**) Tumor weight was measured when nude mice were sacrificed and the weights of xenograft tumours in sh2 group were significantly lower compared to the scr group. p value was calculated by student-t test. (**e,f**) Western blotting analysis detected the TAF15 expression level of xenograft tumours in sh2 group and scr group. (**g–j**) Western blot analysis detected the expression levels of pRAF1/RAF1, pMEK/MEK and pERK/ERK in xenograft tumours of sh2 group and scr group. p value was calculated by student-t test. **p < 0.01, ***p < 0.001, ****p < 0.0001. Three replicates were performed.

To further clarify whether TAF15 contributes to promoting tumourigenesis in vivo, the present study established a xenograft mouse model using HGC27 cells due to their increased tendency to proliferate, migrate and invade. The results demonstrated that knockdown of TAF15 significantly inhibited tumour growth (Fig. 6a,b) and tumour weight (Fig. 6c,d). Furthermore, we identified the level of TAF15 in all xenograft tumours using western blotting analysis. The results showed that the level of TAF15 was significantly reduced in the sh-TAF15 group (sh2) compared to the sh-scrambled group (scr) (Fig. 6e,f). In addition, western blotting analysis further detected the phosphorylation levels of RAF1, MEK and ERK1/2 in 4 random xenograft tumours from the sh2 group and 4 random xenograft tumours from the scr group. The results showed that the phosphorylation levels of RAF1, MEK and ERK were significantly reduced in the sh2 group compared to the scr group, while the total levels of RAF1, MEK and ERK were not significantly changed (Fig. 6g–j). These data indicate that knockdown of TAF15 suppresses tumour growth in vivo via activation of the RAF1/MEK/ERK signalling pathway.

## Discussion

TAF15 was first identified as an RNA or DNA binding protein that was involved in RNAPII-dependent transcription, pre-mRNA splicing, mRNA transport and DNA repair^27^. However, TAF15 has an extended functions beyond RNA or DNA binding. An increasing number of studies have described that TAF15 is also involved in the cellular stress response, cell spreading and cell adhesion^10,28^, which are well known to play a crucial role in tumour cell migration and invasion^29^. Moreover, studies have revealed that TAF15 is associated with the processes of various of malignant tumours including non-small cell lung cancer and melanoma^8,13^. Nevertheless, not much is known about TAF15 in GC. Our study is the first to reveal that TAF15 is significantly upregulated in GC tissues (Fig. 2a,b), and is associated with high T stage in GC patients (Fig. 2c) based on TCGA and TIMER databases. We further confirmed the overexpression of TAF15 in GC tissues and cell lines using western blotting (Fig. 3a–d) and IHC (Fig. 3e,f). Based on the results of IHC analysis, we found that overexpression of TAF15 was positively related to tumour size, T stage and pathologic stage (Table 1). These findings indicated that TAF15 may be an important oncogene with prognostic significance in GC. Interestingly, there was an inconsistent result in the analysis of pathologic stage between immunohistochemical analysis (Table 1, P = 0.0086) and online databases analysis (Fig. 2d, ns) possibly due to the small sample size. In further studies, we will validate this result in larger GC cohorts. Previous studies showed TAF15 knockdown significantly inhibits the proliferation of melanoma and lung cancer cells^8,13^. Similarly, our study verified that TAF15 knockdown suppressed the proliferation (Fig. 4d–h), migration (Fig. 4i–k,o–q) and invasion (Fig. 4l–n) of GC cells in vitro and inhibited tumour growth in vivo (Fig. 6a-d)**.** Indeed, TAF15 can control cell viability via the regulation of the cell cycle and cell death-related genes, as well as specific miRNAs^18^. Therefore, the results of the current study deserve support.

**Figure 7:**
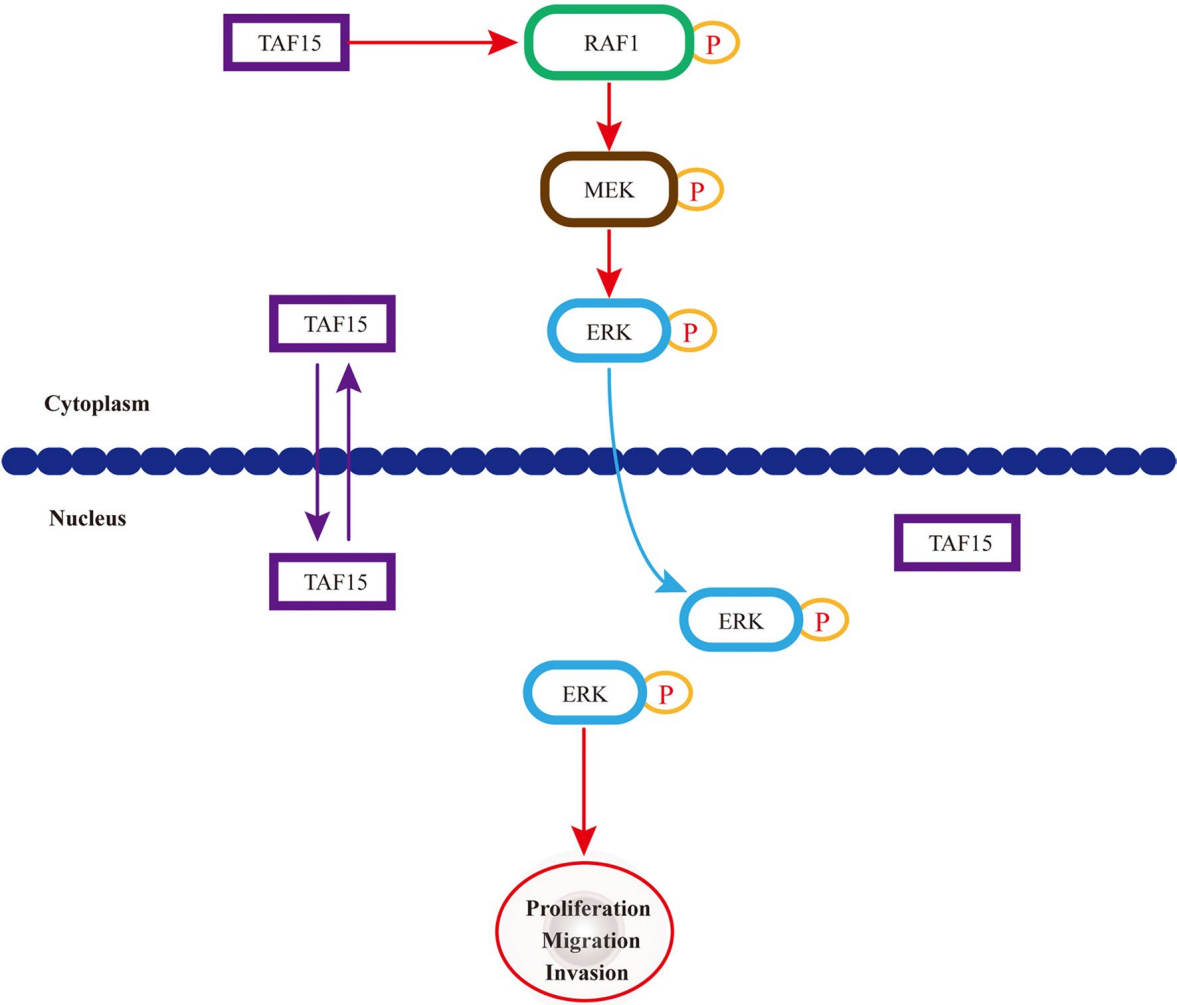
A schematic diagram of the mechanism of TAF15 in GC. TAF15 presents in the cell nucleus and cytoplasm, and promotes the proliferation, migration and invasion of GC cells via the activation of RAF1/MEK/ERK signalling pathway.

The current study also explored the potential molecular mechanism of TAF15 in GC. A previous study found that TAF15 can regulate cell proliferation and the cell cycle by decreasing miR-20a and miR-17-5p and consequently increasing CDKN1A/p21^18^. Moreover, a recent study found that cells expressing EWSR1-FLI1 fusion were certainly positive for pERK1/2 in zebrafish embryos and adult tumours^25^. These studies suggest that the FET family can affect cell biological behaviour through the regulation of a special subset of downstream target genes. However, the potential molecular mechanism of TAF15 in GC is still unknown. Our data first showed that TAF15 knockdown led to a significant decrease in the phosphorylation levels of RAF1, MEK and ERK1/2, while total RAF1, MEK and ERK1/2 exhibited no significant change in GC cell lines (Fig. 5a–h) and xenograft tumours (Fig. 6g–j). Indeed, RAF/MEK/ERK signalling pathway (also known as the MAPK pathway) plays an important role in the regulation of progression in several types of human cancers^26^, and the involvement of this pathway in GC is also widely stated and demonstrated. This pathway can be activated in GC by overexpression of HRC (Histidine-rich calcium binding protein), upregulation of MAGOH and MAGOHB, and overexpression of YAP1 (Yes-associated protein 1), and subsequently promotes the tumour cell proliferation, migration, and invasion^30–32^. These findings indicate that RAF/MEK/ERK pathway plays a crucial role in GC malignant progression. In the current study, we also found overexpression of TAF15 promotes cell proliferation, migration and invasion in GC through activation of the RAF1/MEK/ERK signaling pathway. Similar observations were made in lung squamous cell carcinoma, in which TAF15 was found to promote cell proliferation, migration and invasion by activating the MAPK signaling pathway^20^. Our findings first suggest that TAF15 can regulate the RAF1/MEK/ERK signalling pathway in GC malignant progression (Fig. 7), which greatly expands the functional repertoire of TAF15.

However, the small sample size used for this study was due to the difficulty of collecting paired tumour specimens, which might affect the accuracy of some data. Some findings will be validated in larger GC cohorts. Additionally, other functions of TAF15 remain to be explored, after all, several hundred genes lie downstream of TAF15^18,24^.

## Conclusion

In summary, TAF15 was found to be overexpressed in GC tumour tissues and cells and to contribute to GC malignant progression via the RAF1/MEK/ERK signalling pathway, which suggests that TAF15 might be a potential molecular diagnostic marker or therapeutic target for GC.

## Materials and methods

### Data collection

The Cancer Genome Atlas (TCGA) (https://genome-cancer.ucsc.edu/)^33^ and Tumour Immune Estimation Resource (TIMER) (https://cistrome.shinyapps.io/timer/)^34^ databases are free and accessible data portals with abundant cancer genomes for tumour study. Thus, TCGA and TIMER databases were applied to obtain and analyse RNA-Seq expression and clinical pathologic data of GC patients.

### Tissue samples

One hundred thirty-one GC patients from Yantai Yuhuangding Hospital of Qingdao University were recruited from November 22, 2021 to March 17, 2022 in the current research. All patients were diagnosed with GC by two pathologists according to the standard of diagnosis and received no radiotherapy or chemotherapy before surgery. Among the 131 samples, 24 samples had tumour tissues (T) and matched adjacent normal tissues (N), while 107 samples had only tumour tissues. All samples were frozen in liquid nitrogen within 30 min postoperatively. The clinical information of all patients is documented in Supplemental Table S1.

### Western blotting

Twenty-four paired GC tissues and cell lines (MKN28, AGS, MKN45, HGC27 and GES-1) were lysed in RIPA lysis buffer containing 1% protease inhibitors. The lysates of GC tissues and cell lines were centrifuged at 12,000 × *g* for 10 min at 4 °C twice, and then the supernatants were transferred to new tubes. The protein concentration was quantified using a BCA assay kit (catalogue no.P0010, Beyotime Biotechnology, Shanghai, China). All protein samples (25 µg/load) were separated by using 10% sodium dodecyl sulfonate-polyacrylamide gel electrophoresis (SDS-PAGE) and transferred onto polyvinylidene difluoride (PVDF) membranes. Whereafter, PVDF membranes were blocked for 1 h with 5% nonfat milk in TBST buffer at room temperature and incubated with primary antibodies overnight at 4 °C. The next day, membranes were incubated in secondary antibodies at room temperature for 1 h. Finally, PVDF membranes were developed by ECL reagents (MA0186, Meilunbio, Dalian, China) through a ChemiScope 6200 Touch Imaging System (CLINX, Shanghai, China). In addition, some of the membranes were incubated with two or more primary antibodies repeatedly by using stripping buffer (SL1340, Coolaber, Beijing, China). The primary antibodies were applied to this study as follows: TAF15 (1:3000, ab134916, Abcam, USA), GAPDH (1:5000, D110016, Sangon Biotech, Shanghai, China), RAF1 (1:500, D155090, Sangon Biotech, Shanghai, China), p-RAF1 (1:1000, 66,592-1-lg, Proteintech, Wuhan, China), MEK1/2 (1:1000, 11,049-1-AP, Proteintech, Wuhan, China), p-MEK1 (1:1000, 28,930-1-AP, Proteintech, Wuhan, China), ERK1/2 (1:1000, 16,443-1-AP, Proteintech, Wuhan, China), and p-ERK1/2 (1:1000, 28,733-1-AP, Proteintech, Wuhan, China). The secondary antibodies used were as follows: HRP goat anti-rabbit IgG (1:5000, D110058, Sangon Biotech, Shanghai, China), and HRP goat anti-mouse IgG (1:5000, D110058, Sangon Biotech, Shanghai, China).

### Immunohistochemistry (IHC)

One hundred thirty-one GC tissues and twenty-four matched adjacent normal tissues were embedded in paraffin according to standard procedures. All tissue blocks were cut into 4 µm sections and then deparaffinized and hydrated on the basis of standard procedures. The endogenous peroxidase activity of tissue sections was blacked by using 3% H_2_O_2_ for 15 min at room temperature. Sections were incubated in a wet chamber with the primary antibody TAF15 (1:100, ab134916, Abcam, USA) at 4 °C for overnight, and then incubated with the secondary antibody HRP goat anti-rabbit IgG (1:100, D110058, Sangon Biotech, Shanghai, China) for 1 h at room temperature. Finally, sections were developed using diaminobenzidine and haematoxylin. The images were reviewed by two pathologists in a blinded fashion. The staining percentage was scored as follows: 0 (< 5% positive cells); 1 (5–24% positive cells); 2 (25–49% positive cells); 3 (50–74% positive cells) and 4 (≥ 75% positive cells). The staining intensity was scored as follows: 0 (no staining), 1 (weak), 2 (moderate), and 3 (strong). The combined staining score was equivalent to the staining percentage score × staining intensity score and a combined score ≥ 4 was defined as positive expression, while a combined score < 4 was considered negative expression.

### Cell culture and cell transfection

MKN28, AGS, MKN45, HGC27 and GES-1 cell lines were purchased from the China Academy of Chinese Medical Sciences. MKN28, MKN45, HGC27 and GES-1 cells were cultured in RPMI-1640 medium (BioInd, Israel) and AGS cells were cultured in DMEM (BioInd, Israel) with 10% fetal bovine serum (FBS) (BioInd, Israel) and 1% penicillin–streptomycin in a humidified 37 °C incubator with 5% CO2. The lentivirus for knocking down TAF15 (shRNA1, shRNA2, sh-scrambled) was obtained from Genomeditech (Shanghai, China). The sequences of shRNA were as follows: shRNA1: GGAGAAGATAATAGAGGATAT; shRNA2: GGGTGTGTCTACAGATCAAGT; sh-scrambled: TTCTCCGAACGTGTCACGT.AGS and HGC27 cells were cultured in 12-well plates (Corning Life Sciences) at 1 × 10^5^ cells per well. Cell transfection was executed when cell density get to about 50%. After being transfected for 48 h, the cells were cultured in fresh medium. The Multiplicity of infection of lentivirus in AGS and HGC27 cells was 20. Stably transfected cell lines were selected using puromycin (1 μg/mL). The transfection efficiency was detected by western blotting analysis.

### CCK8 and colony formation assays

Three groups of stably transfected AGS and HGC27 cells (shRNA1, shRNA2, sh-scrambled) were seeded at 1 × 10^3^ cells per well into 96-well plates and cultured for 24 h, 48 h, 72 h, and 96 h. During the last hour, Cell Counting Kit-8 (CCK-8) reagent (Dojindo, Tokyo, Japan) (10 µl/well) was added to the individual wells. A microplate reader (Bio-Rad) was used to measure the absorbance at 450 nm. For the colony formation assay, stably transfected AGS and HGC27 cells (shRNA1, shRNA2, sh-scrambled) were inoculated into 6-well plates (500 cells/well) and cultured for 10 days. Colonies were fixed with 4% paraformaldehyde for 30 min, and stained with 1% crystal violet for 15 min. Finally, colonies comprising 50 or more cells were counted. All experiments were repeated three times.

### Transwell experiment

Stably transfected AGS and HGC27 cells (shRNA1, shRNA2, sh-scrambled) were inoculated at 2 × 10^4^ cells per well into the upper polycarbonate membrane chambers without FBS, while medium containing 10% FBS was added to the lower chamber. When the invasion test was performed, matrix gel was added to the upper chambers beforehand. After 48 h of culture, the cells were removed from the lower side of the compartment, fixed for 30 min by using 4% paraformaldehyde, and then stained with 1% crystal violet for 15 min. Finally, images were taken, and five random fields were selected for counting by using an inverted microscope. The experiment was performed three times.

### Wound healing assay

Stably transfected AGS and HGC27 cells (shRNA1, shRNA2, sh-scrambled) were cultured in a 6-well plate until 90% confluent, and the cell monolayer of each well was scratched using a 1 ml plastic pipette tip. The cell debris was washed away twice with culture medium and the cells were cultured in medium with 10% FBS. Took photographs at 0 h and 36 h after scratching using an Olympus microscope. The experiment was performed three times. The rate of wound healing was evaluated using ImageJ software.

### Animal model experiment

Four- to five-week-old female nude mice were obtained from the Beijing Vital River Laboratory Animal Technology Co., Ltd (Beijing, China) and fed under pathogen-free conditions. Nude mice were randomly divided into two groups (10 mice per group), after which stably transfected sh-TAF15 or sh-scrambled cells (2 × 10^6^ cells per mouse) were subcutaneously injected into nude mice in the armpit. The xenograft tumour volumes (volume = length × width × width/2) of nude mice were measured every day. After 26 days of injection, all mice were sacrificed by using the cervical dislocation method under anaesthesia (0.7% sodium pentobarbital). Then, xenograft tumours were excised, photographed and weighed. A double-blind method was executed for this experiment.

### Statistical analysis

The data of the current study were statistically analysed by using GraphPad Prism 9.3.1. Student’s t test, chi-square test, one-way analysis of variance (ANOVA), Mann–Whitney U test and Kruskal–Wallis test were performed to analyse the data. The results are presented as the mean ± standard deviation (SD). P < 0.05 was considered statistically significant.

### Ethics declarations

This study was performed in line with the principles of the Declaration of Helsinki and approved by the research medical ethics committee of Yantai Yu Huang Ding Hospital of Qing Dao University (No.2022–46). All methods were performed in accordance with the relevant guidelines and regulations. All patients signed the informed consent form. This study followed the recommendations in the ARRIVE guidelines.

## Supplementary Information

Supplementary Figures.

Supplementary Table S1.

## Data Availability

All datasets generated or analysed during the current study are included in this published article.
