# Supplementary material for: TAF15 promotes cell proliferation, migration and invasion of gastric cancer via activation of the RAF1/MEK/ERK signalling pathway
Source: Sci Rep. 2023 Apr 10;13:5846. doi: 10.1038/s41598-023-31959-0 (PMC10086039; doi:10.1038/s41598-023-31959-0)
Supplement: Supplementary file 1 — Supplementary Figures. [file 41598_2023_31959_MOESM1_ESM.pdf]

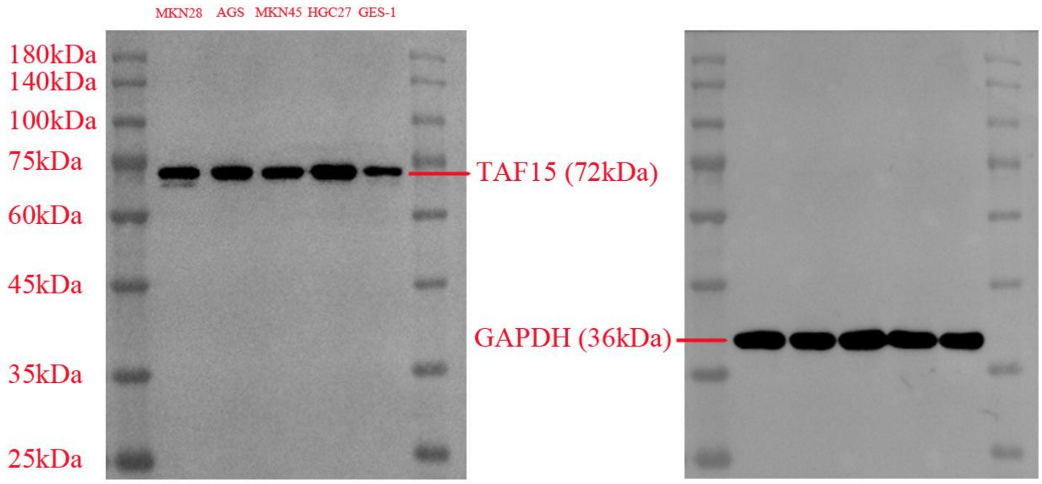

Original figure 1 match to Figure 3a

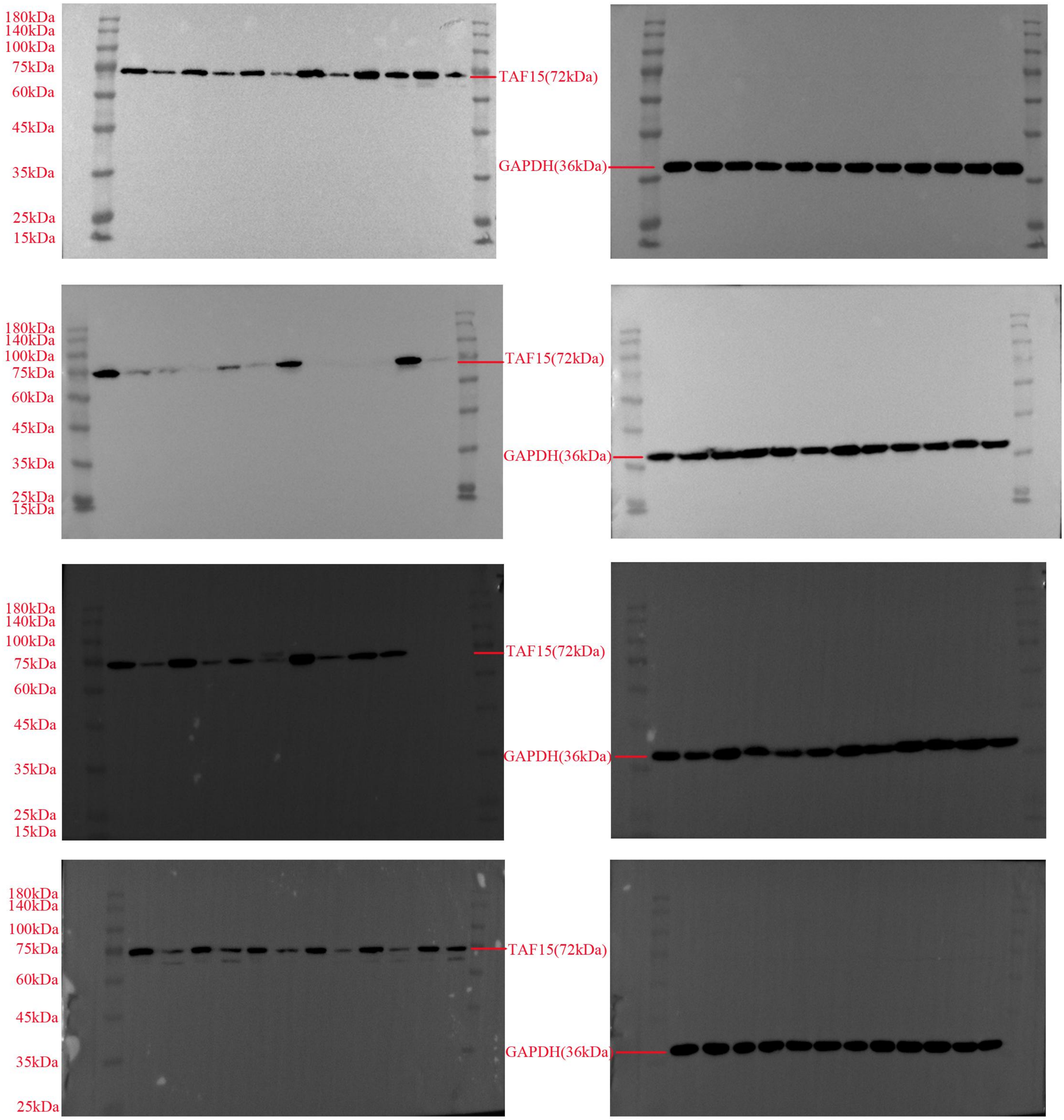

Original figure 2 match to Figure 3c

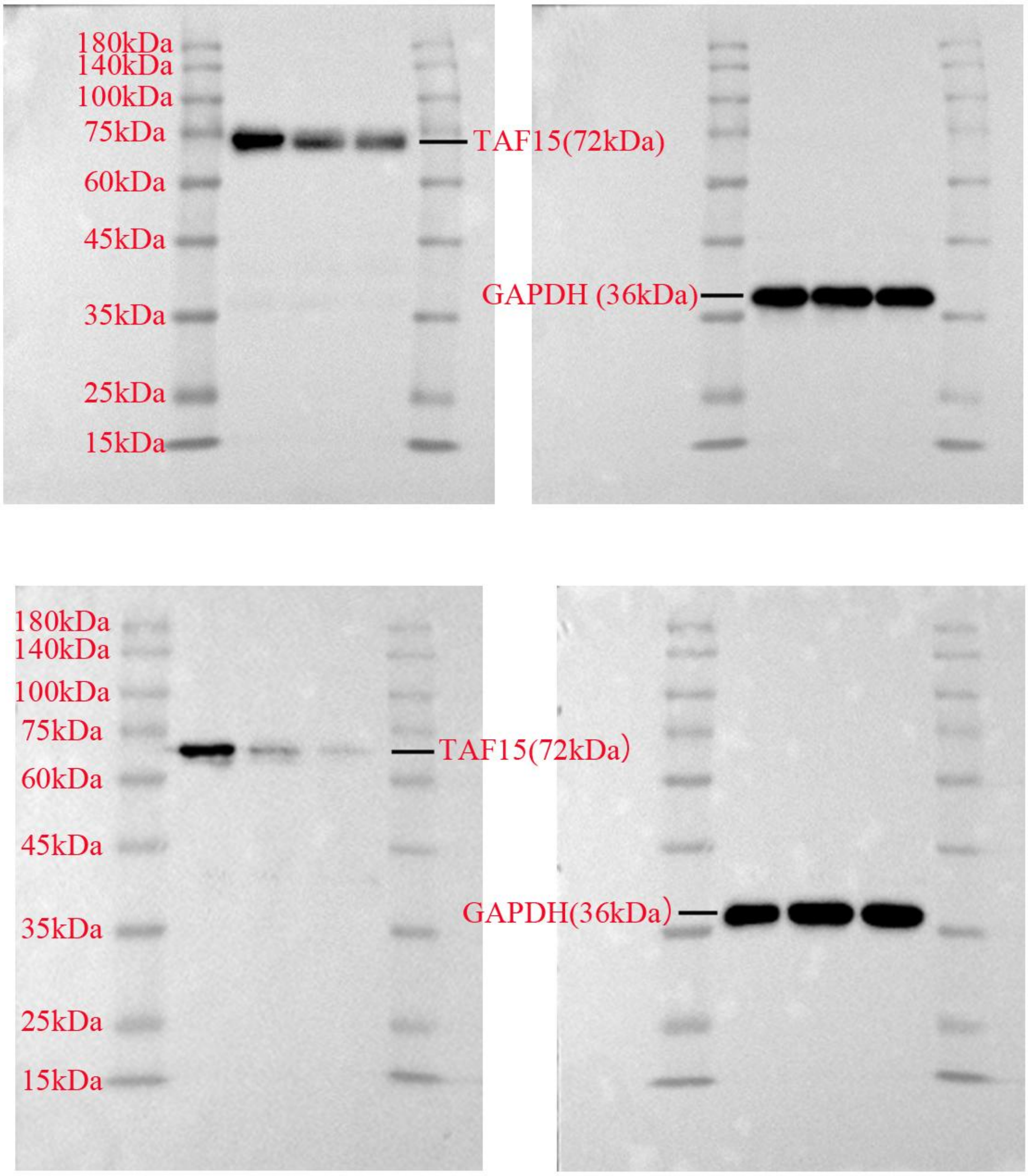

Original figure 3 match to Figure 4a

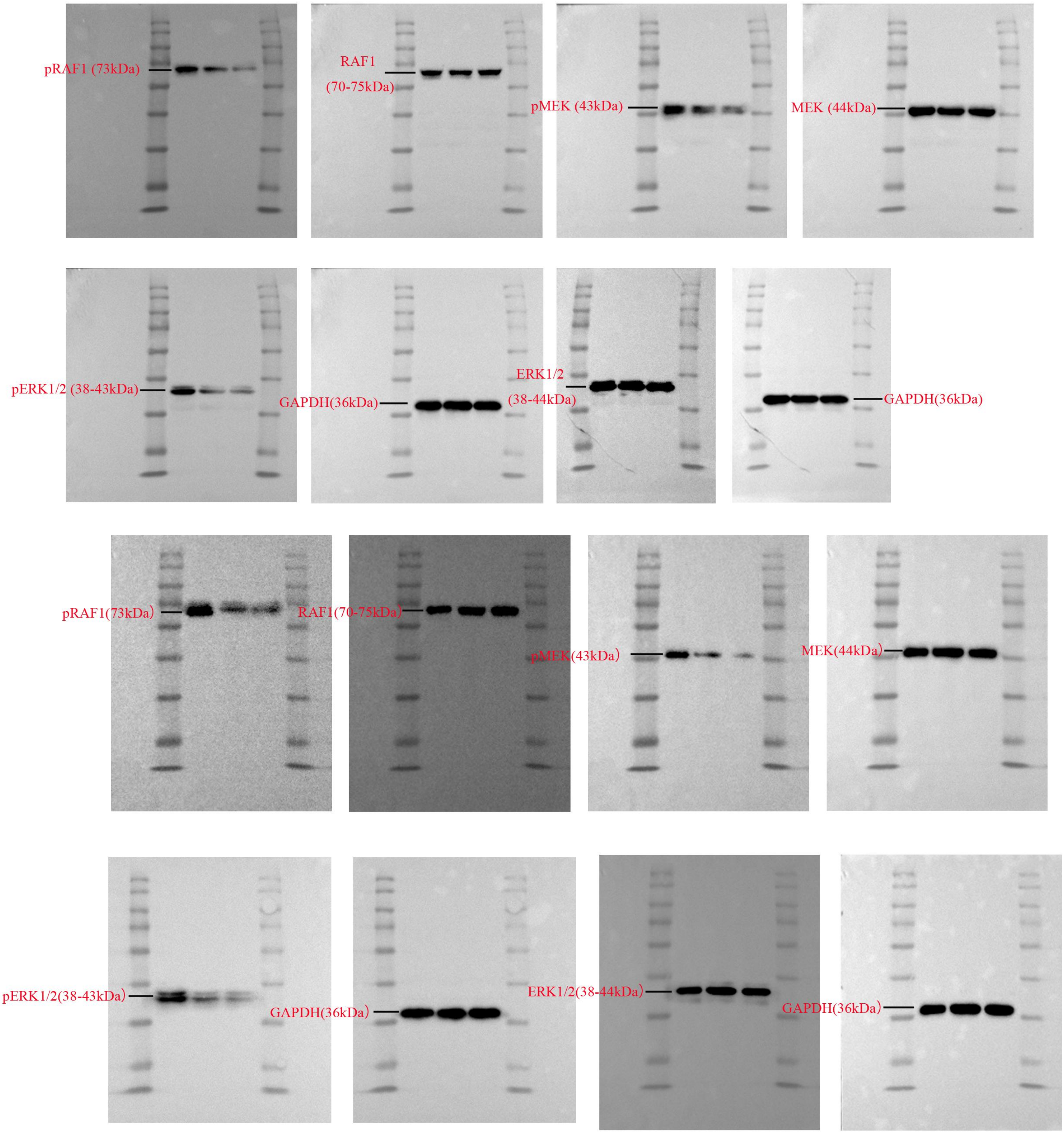

Original figure 4 match to Figure 5a, e

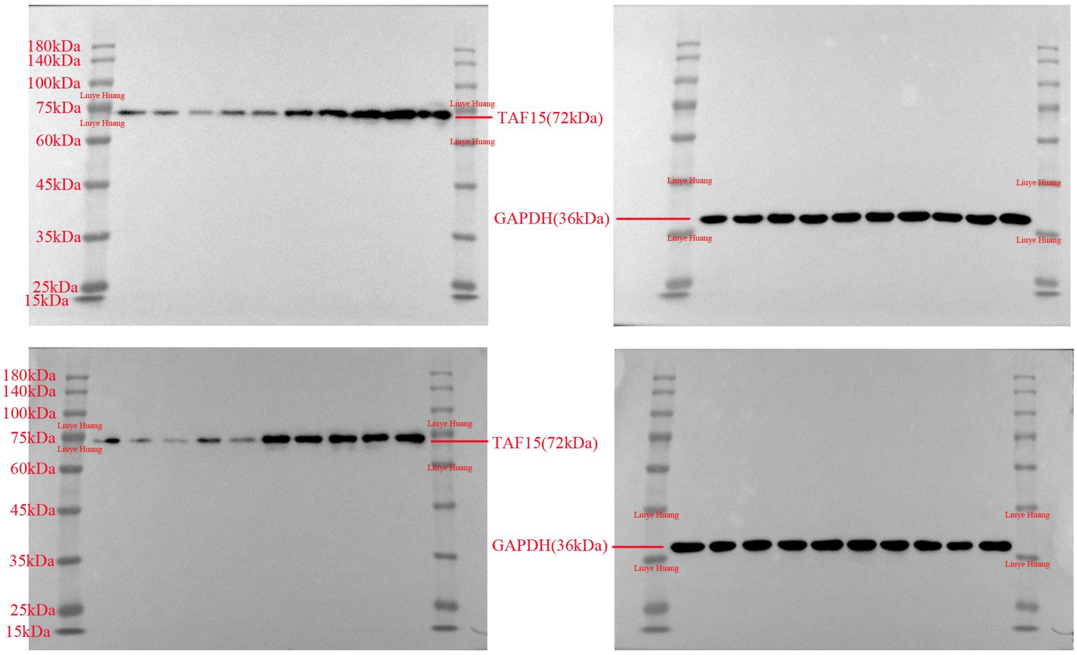

Original figure 5 match to Figure 6e

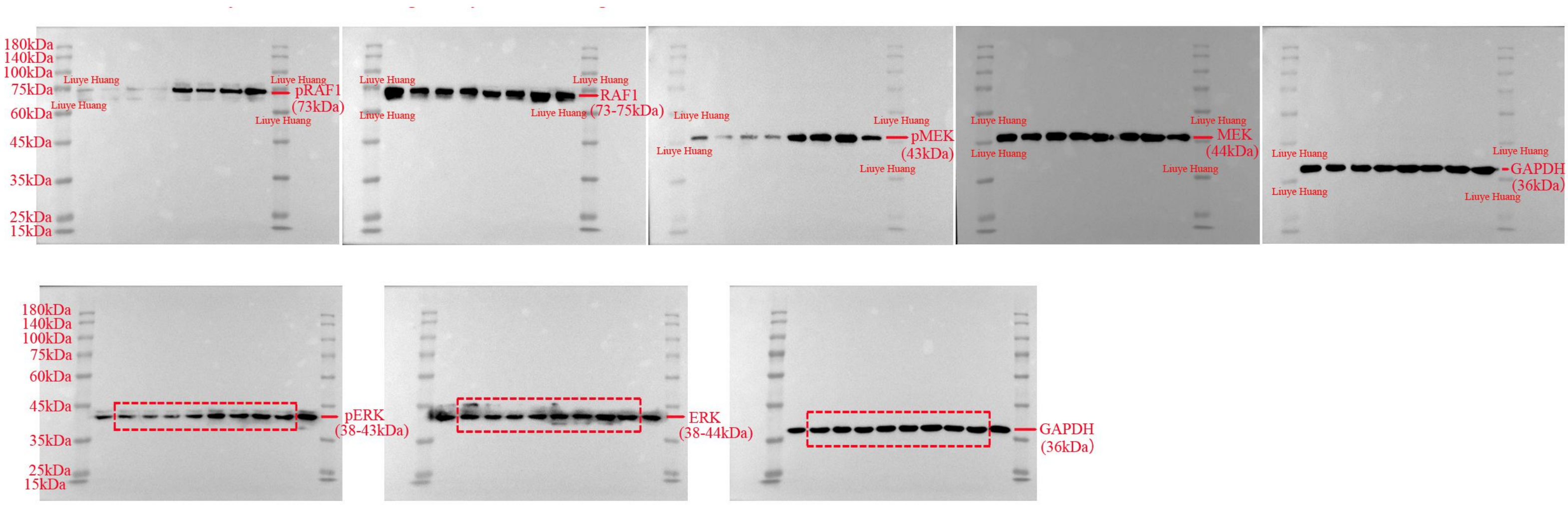

Original figure 6 match to Figure 6g
